# Intraoperative Airway Obstruction: A Case Report

**DOI:** 10.7759/cureus.91089

**Published:** 2025-08-27

**Authors:** Muhammad Usman, Tomohiro Yamamoto, Satoshi Yamamoto

**Affiliations:** 1 Anesthesiology, University of Texas Medical Branch at Galveston, Galveston, USA; 2 Department of Medicine, Gunma University School of Medicine, Maebashi, JPN

**Keywords:** airway obstruction, clinical case report, difficult airway management, intratracheal hemorrhage, video bronchoscopy

## Abstract

Intraoperative airway obstruction due to acute intratracheal hemorrhage represents a rare yet life-threatening anesthetic emergency requiring immediate recognition and urgent intervention for critical airway obstruction. We present the case of a 66-year-old male with extensive medical comorbidities undergoing rib plating for traumatic fractures, who experienced sudden absence of end-tidal CO₂ and bloody secretions following elective exchange from a single-lumen to a double-lumen endotracheal tube. Emergent bronchoscopy identified extensive obstructing tracheobronchial blood clots. Initial management with a supraglottic airway provided temporary oxygenation but proved insufficient for clot removal. Definitive airway clearance required intervention involving rigid bronchoscopy with optical forceps and cryoextraction. Subsequent imaging and ENT evaluation identified minor mucosal trauma as the likely bleeding source, exacerbated by the patient's underlying coagulopathy. Following controlled airway management and intensive care monitoring, the patient was successfully extubated and recovered fully without further complications.

## Introduction

The period immediately preceding and during surgical airway manipulation presents distinct challenges for anesthesiologists and surgeons, typically accentuated by rapid physiological variations and unexpected problems. Among the various difficulties, airway blockage presents a notably tough situation because it can endanger oxygen flow and ventilation almost instantly, leaving little margin for error [1]. The etiology of intraoperative airway obstruction is multifactorial, incorporating mechanical factors such as the displacement or occlusion of airway devices, aspiration of extraneous materials, and, as exemplified in this case, hemorrhage resulting in luminal obstruction.

Although uncommon, airway obstruction due to tracheobronchial bleeding and clot can be life-threatening and demands immediate recognition and intervention [2]. Early manifestations may be subtle, such as rising airway pressures or unexplained desaturation, but can rapidly progress to catastrophic loss of ventilation, including an abrupt absence of capnography with visible blood in the airway [3]. Delayed diagnosis or response risks hypoxia, cardiovascular collapse, and death [4].

Patients with complicated medical presentations, including those with coagulopathy, such as in cirrhosis, are especially vulnerable to bleeding complications, highlighting the importance of targeted preoperative risk assessment and planning [5]. Contemporary difficult-airway guidelines further emphasize thorough preoperative evaluation and the formulation of primary and backup strategies [6]. In addition, efficient interdisciplinary coordination among anesthesiologists, surgeons, otolaryngologists, and interventional pulmonologists is critical for timely diagnostic clarification and definitive airway control [7].

Advances in airway management have broadened therapeutic options. Videolaryngoscopy improves first-attempt success and reduces failed intubation compared with direct laryngoscopy in adults [8]. Therapeutic bronchoscopy, including rigid and flexible bronchoscopy, provides large-bore access for suction and instrument deployment when confronting central airway obstruction [9]. In addition, specialized extraction tools such as cryoprobes enable en bloc removal of tracheobronchial clots when conventional suction is ineffective [10]. Ultimately, successful outcomes rely not only on the availability of these modalities but also on team readiness and coordinated execution.

In this report, we describe the rapid onset and resolution of a life-threatening intraoperative airway obstruction from acute intratracheal hemorrhage with clot formation, with attention to the diagnostic sequence and procedural strategies that supported a favorable outcome. We hope our strategy proves useful for future patients facing similar risks.

## Case presentation

A 66-year-old male with a history of infective endocarditis, mitral valve perforation, cirrhosis, embolic stroke, and multiple chronic comorbidities was scheduled for rib plating after sustaining multiple left-sided rib fractures due to a ground-level fall. The anesthetic plan included general anesthesia with endotracheal intubation, arterial line placement, and bronchial blocker. The patient was initially intubated effectively utilizing a 7.5 mm endotracheal tube. A left-sided double-lumen tube was then selected to achieve lung isolation and one-lung ventilation, providing optimal surgical exposure and protecting the contralateral lung during the procedure. Subsequent to the flexible bronchoscopy performed by the surgical team, the endotracheal tube was replaced with a 39 Fr. left double-lumen endotracheal tube over a bougie via a Cook exchanger with the aid of direct laryngoscopy. Although the exchange was executed smoothly and appeared atraumatic, and both the sampling line and ventilatory circuit remained intact, end-tidal CO₂ (ETCO₂) was noted to be absent. Despite adjusting the depth of the double-lumen tube, it was concluded to take it out, and a supraglottic airway was then utilized. At this particular point in time, in addition to the absence of ETCO₂, the presence of sanguineous secretions observed within the oropharyngeal region was documented.

Emergent bronchoscopy revealed extensive blood clots obstructing the trachea and bilateral mainstem bronchi, prompting urgent otolaryngology and interventional pulmonology consultation. Rigid bronchoscopy with optical forceps and cryoprobe extraction was performed, successfully removing the clots and partially restoring ventilation. The airway was ultimately secured with an 8.0 endotracheal tube under a glidescope. The examination of the airway that includes the oropharyngeal region, trachea, and bronchi, performed with a fibroscope, has currently indicated that no visible source of bleeding was found. While the oxygen levels of the patient did eventually stabilize to normal following the preoxygenation procedure, the ongoing circulation issues, together with extended drops in oxygen saturation, caused the decision to halt the procedure; hence, the patient was transported to the Surgical Intensive Care Unit (SICU) while undergoing intubation and sedation for ongoing airway management. Contrast-enhanced computed tomography was assessed for potential hemorrhagic sources, yielding negative results. The patient exhibited effective communication following the cessation of sedation within the intensive care unit. Subsequent otolaryngology operative evaluation revealed ecchymoses of the palate and tonsillar fossae, a narrowed cricoid variant, mucosal abrasion in the right upper segmental bronchus, and pressure injury to the vocal folds. No mucosal tears or significant structural airway injury were identified. Under meticulously regulated circumstances, the process of extubation was executed with success. The patient was transferred to the intensive care unit for recovery. 

Laboratory data for the patient are described below. Complete blood count with differential obtained on the day of the event and repeated the following day is summarized in Table 1. 

**Table 1:** Complete blood count (CBC) before and after an event WBC, white blood cell count (×10³/µL); RBC, red blood cell count (×10⁶/µL) (low); HGB, hemoglobin (g/dL) (low); HCT, hematocrit (%) (low); MCV, mean corpuscular volume (fL); MCH, mean corpuscular hemoglobin (pg); MCHC, mean corpuscular hemoglobin concentration (g/dL); RDW-SD, red cell distribution width-standard deviation (fL); RDW-CV, red cell distribution width-coefficient of variation (%); PLT, platelet count (×10³/µL); MPV, mean platelet volume (fL) (low)

Lab	Before event	After event	Reference ranges
WBC ×10^3/µL	5.09	6.12	4.2–10.7
RBC ×10^6/µL	3.81	3.35	4.3–5.5
HGB (g/dL)	10.7	10.1	12.2–16.4
HCT (%)	31.4	29.7	38.4–49.3
MCV (fL)	82.4	88.7	81.7–95.6
MCH (pg)	28.1	30.1	26.1–32.7
MCHC (g/dL)	34.1	34.0	31.2–35.0
RDW-SD (fL)	44.3	45.8	38.5–51.6
RDW-CV (%)	14.7	14.3	12.1–15.4
PLT ×10^3/µL	230	271	150–328
MPV (fL)	8.8	9.1	9.8–13.0

The basic metabolic panel (BMP) obtained on the day of the event and repeated the following day is presented in Table 2. The comparative display demonstrates peri-event electrolyte and acid-base shifts pertinent to the airway emergency and resuscitation.

**Table 2:** Basic metabolic panel before and after event Na, sodium (mmol/L) (low after event); K, potassium (mmol/L); Cl, chloride (mmol/L) (low after event); CO2 total, total carbon dioxide/bicarbonate (mmol/L) (low after event); anion gap (mmol/L); BUN, blood urea nitrogen (mg/dL) (low after event); creatinine (mg/dL); glucose (mg/dL) (high); calcium (mg/dL) (low after event); eGFR, estimated glomerular filtration rate (mL/min/1.73 m²)

Lab	Before event	After event	Reference ranges
Na (mmol/L)	135	130	135–145
K (mmol/L)	5	5	3.5–5.0
Cl (mmol/L)	101	97	98–108
CO2 total (mmol/L)	30	20	23–31
Anion gap (mmol/L)	4	13	2–16
BUN (mg/dL)	23	14	7–23
Glucose (mg/dL)	147	176	70–110
Creatinine (mg/dL)	0.85	0.72	0.6–1.3
Calcium (mg/dL)	8.6	7.7	8.6–10.2
eGFR (mL/min/1.73 m²)	95.8	100.8	≥60

Arterial blood gas/co-oximetry with lactic acid. Obtained two days post event, these results are summarized in Table 3. The data show adequate oxygenation with normal lactate and mild alkalemia with slightly elevated PaCO₂, consistent with post-event recovery. Lower oxygen content in labs likely reflects persistent anemia rather than impaired gas exchange.

**Table 3:** Arterial blood gas/co-oximetry with lactic acid 2 days after event pH (high); PCO₂, partial pressure of carbon dioxide (mmHg); PO₂, partial pressure of oxygen (mmHg) (high); HCO₃⁻, bicarbonate (mEq/L) (high); Base excess (mEq/L) (high); tHb, total hemoglobin (g/dL) (low); %O₂Hb, oxyhemoglobin (%); %COHb, carboxyhemoglobin (%); %MetHb, methemoglobin (%) (low); O₂ content vol%, oxygen content (vol%, mL O₂/dL) (low); Na, sodium (mmol/L); K, potassium (mmol/L); Ionized Ca, ionized calcium (mg/dL); glucose (mg/dL) (high); lactic acid (mmol/L)

Lab	Result	Reference ranges
pH	7.5	7.35–7.45
PCO₂ (mmHg)	35	35–45
PO₂ (mmHg)	136	80–100
HCO₃⁻ (mEq/L)	27	22–26
Base excess (mEq/L)	3.7	−3–3
tHb (g/dL)	10.4	13.5–18
%O₂Hb	98.2	94–99
%COHb	0.5	0.0–1.5
%MetHb	0.3	0.4–1.5
O₂ content, vol% (mL O₂/dL)	14.6	15.0–23.0
Na (mmol/L)	139	135–145
K (mmol/L)	4.1	3.5–5.0
Ionized Ca (mg/dL)	4.6	4.5–5.3
Glucose (mg/dL)	139	70–110
Lactic acid (mmol/L)	1.31	0.5–2.2

Post-event coagulation studies are summarized in Table 4; pre-event values were not available. These results provide context for bleeding risk assessment and procedural decision-making during airway management.

**Table 4:** Coagulation studies after event PT(s), prothrombin times; INR, international normalized ratio; aPTT(s), activated partial thromboplastin times; fibrinogen (mg/dL) (high)

Lab	Results	Reference ranges
PT(s)	11.3	10.1–12.6
INR	1	<1.1
aPTT(s)	36	26–36
Fibrinogen (mg/dL)	716	167–453

## Discussion

This case highlights the distinctive hazards of intraoperative airway obstruction precipitated by acute hemorrhage and adherent thrombus, an uncommon but recognized scenario requiring careful planning and rapid action [2]. The patient’s comorbidities, particularly cirrhosis-associated coagulopathy, likely increased bleeding risk despite an apparently atraumatic tube exchange [5]. Intraoperative transitions from a single-lumen to a double-lumen endotracheal tube can injure fragile airway mucosa and provoke hemorrhage, especially in patients with bleeding diatheses or abnormal airway anatomy [11]. The immediate loss of end-tidal CO₂ after the exchange, accompanied by sanguineous secretions, signaled critical obstruction and required urgent intervention [3].

Initial management appropriately prioritized restoration of oxygenation while the team identified and relieved the obstruction. A supraglottic airway can temporarily maintain oxygen delivery but cannot bypass or resolve distal tracheal obstruction from a clot [12]. Flexible bronchoscopy is invaluable diagnostically; however, visualization is frequently obscured during active bleeding, and large, organized clots often resist removal with standard suction or forceps alone [10].

Definitive airway clearance was achieved with rigid bronchoscopy, which provided stable airway control, large-bore suction, and a wide working channel for therapeutic instruments in central airway obstruction [9]. Adjunctive cryoextraction was then used to freeze-adhere to the organized clot and remove it en bloc, a technique shown to be effective and safe in hemorrhagic airway obstruction [13]. The success of this combined approach shows the value of institutional preparedness. Owing to ready access to advanced bronchoscopic tools, clinicians skilled in their use, as well as the coordinated collaboration among the responding teams, the patient was able to recover.

Post-intervention inspection revealed only minor mucosal trauma without an ongoing bleeding source. Given the documented complication profile after therapeutic bronchoscopy, careful post-procedure assessment is important to mitigate risks of rebleeding, airway injury, or stenosis [14]. The decision to abort the planned operation and proceed with intensive care monitoring is in line with best practices in the management of life-threatening airway hemorrhage, where airway protection, hemodynamic stabilization, and stepwise hemostatic control take precedence [4].

Broader implications include the need for tailored preoperative risk stratification and contingency planning in patients at heightened bleeding risk, whether due to anticoagulant therapy, hepatic dysfunction, or atypical airway anatomy. Guidance statements for periprocedural hemostasis in cirrhosis recommend individualized assessment and targeted correction rather than reflexive normalization of conventional coagulation tests [15]. Finally, written protocols and team-based rehearsal, coupled with early, structured multidisciplinary involvement, facilitate rapid deployment of equipment and skills when airway emergencies arise [7].

## Conclusions

This case shows the importance of early recognition, rapid response, and institutional preparedness when an acute intratracheal hemorrhage with clot formation compromises the airway. A structured approach, which prioritizes oxygenation, prompt bronchoscopic diagnosis, and definitive clearance with rigid bronchoscopy, can turn a potentially fatal event into a controlled perioperative crisis. Equally important are preoperative risk stratification for bleeding, clear contingency plans for airway rescue, and vigilant postoperative monitoring to detect and manage delayed complications, all of which help minimize patient harm during intraoperative emergencies.
